# Andrographolide binds to ATP-binding pocket of VEGFR2 to impede VEGFA-mediated tumor-angiogenesis

**DOI:** 10.1038/s41598-019-40626-2

**Published:** 2019-03-11

**Authors:** Kirti Kajal, Abir K. Panda, Jyotsna Bhat, Dwaipayan Chakraborty, Sayantan Bose, Pushpak Bhattacharjee, Tania Sarkar, Subhrangsu Chatterjee, Santosh K. Kar, Gaurisankar Sa

**Affiliations:** 1 Division of Molecular Medicine, Bose Institute, P-1/12, Calcutta Improvement Trust Scheme VII M, Kolkata, 700054 India; 2 Department of Biophysics, Bose Institute, P-1/12, Calcutta Improvement Trust Scheme VII M, Kolkata, 700054 India; 3 School of Biotechnology, KIIT University, Bhubaneswar, 751024 India

## Abstract

Vasculogenesis and angiogenesis are process of formation of blood vessels. Blood vessels are evolved to distribute nutrients and oxygen to distant organs. These vessels are crucial for growth and repair of wounded tissue. During tumor condition there occurs imbalance in the growth of blood vessels which leads to neo-angiogenesis. Neo-angiogenesis is major perpetrator behind the establishment of tumor. Tumor cells secrete pro-angiogenic factor VEGFA which binds to VEGFR2 present over surface of endothelial cells and triggers formation of new blood vessels. To inhibit tumor-angiogenesis, a physiologically-safe small molecule inhibitor was screened which can potentially interact with kinase domain of VEGFR2 and inhibit its activity. Molecular-docking module and biochemical analysis identified andrographolide as one of the best docking molecules that binds to ATP-binding pocket of VEGFR2 and inhibits its kinase activity. Thus, for a more radical approach towards safe VEGFR2 inhibitor, andrographolide was repurposed to inhibit tumor-angiogenesis and reduce tumor burden.

## Introduction

After an era of intense research, cancer is still a major area of concern. It has the capacity to survive in all conditions and paralyze host system^1^. New blood vessel formation or angiogenesis in the tumor microenvironment is known as tumor-angiogenesis/neo-angiogenesis which is one of the major *Hallmark of Cancer*^2^. Judah Folkman first coined the term “tumor angiogenesis”^3^. Angiogenesis is a controlled series of event which involves the formation of new blood vessels from pre-existing vessels causing neovascularization^4^. Under normal condition angiogenesis occurs during development, vascular remodeling, wound healing, pregnancy, menstrual cycle while in the pathological condition it occurs during growth of malignant tumors^4,5^. There is always a balance between pro- and anti-angiogenic signals, and this balance gets disrupted under tumor condition. When tumor size exceeds beyond a defined size; lack of blood and oxygen supply and nutrient deficit condition prompts angiogenic-switch from vascular quiescence^6^. This angiogenic-signal leads to the release of vascular endothelial growth factor (VEGFA), bFGF, EGF, cytokines and chemokines.

VEGF binds to vascular endothelial growth factor receptor (VEGFR) present on the surface of endothelial cells and initiates neo-angiogenesis. Amongst receptor protein tyrosine kinases - VEGFR1 (FLT1), VEGFR2 (Flk-1/KDR) and VEGFR3, its VEGFR2 which plays significant role in neo-angiogenesis. Structure of VEGFR2 comprises an extracellular domain (seven immunoglobulin-like segments), a short membrane-spanning region, a juxtamembrane regions, cytosolic tyrosine-kinase domain and a carboxy terminal tail. Binding of VEGF with VEGFR2 leads to protein tyrosine kinase activation leading to the activation of downstream signaling molecules which mediates endothelial cell migration, proliferation, survival, and vascular permeability^7,8^.

Hence, to combat tumor-angiogenesis, we need to inhibit VEGFR2 activation. Commonly known tyrosine kinase inhibitors *viz*. sunitinib, sorafenib, pazopanib, bevacizumab, axitinib and tivozanib are used in clinic to inhibit VEGFR2. They are nonspecific, cause fatigue, asthenia, diarrhea, stomatitis, skin toxicities and several cardiovascular diseases^9^. At this juncture, there is an utmost necessity to search for specific high-affinity VEGFR2 inhibitor which is non-toxic and has high bioavailability. A preliminary search indicates that the well-known small molecule, andrographolide, specifically binds to the kinase domain of VEGFR2 and inhibits its kinase activity. The herb *Andrographis paniculata*, the source of andrographolide is obtained in the Indian subcontinent^10^. It attenuates inflammation by inhibiting NFκB-mediated pro-inflammatory cytokines production^11^. Recently it was observed that andrographolide prevent bone loss by suppressing RANKL-mediated NFκB and ERK-signaling that lead to human breast cancer-induced osteoclast differentiation^12^. Shen *et al*. have reported that andrographolide has the ability to inhibit angiogenesis^13^. Some questions need to be answered yet, (i) Mode of action behind andrographolide mediated VEGFR2 inactivation. More importantly, whether the non-toxic andrographolide can suppress VEGFA-mediated neo-angiogenesis? Finally, is it efficient enough to regress tumor load in mice tumor model? To unravel this mystery, the study was designed to repurpose this anti-inflammatory, hepatoprotective drug^10^ in annulling tumor-angiogenesis and diminishing tumor burden in the host.

## Results

### Andrographolide and tivozanib, a known inhibitor of VEGFR2, interacts with common amino acid in the ATP-binding pocket of VEGFR2

**Figure 1:**
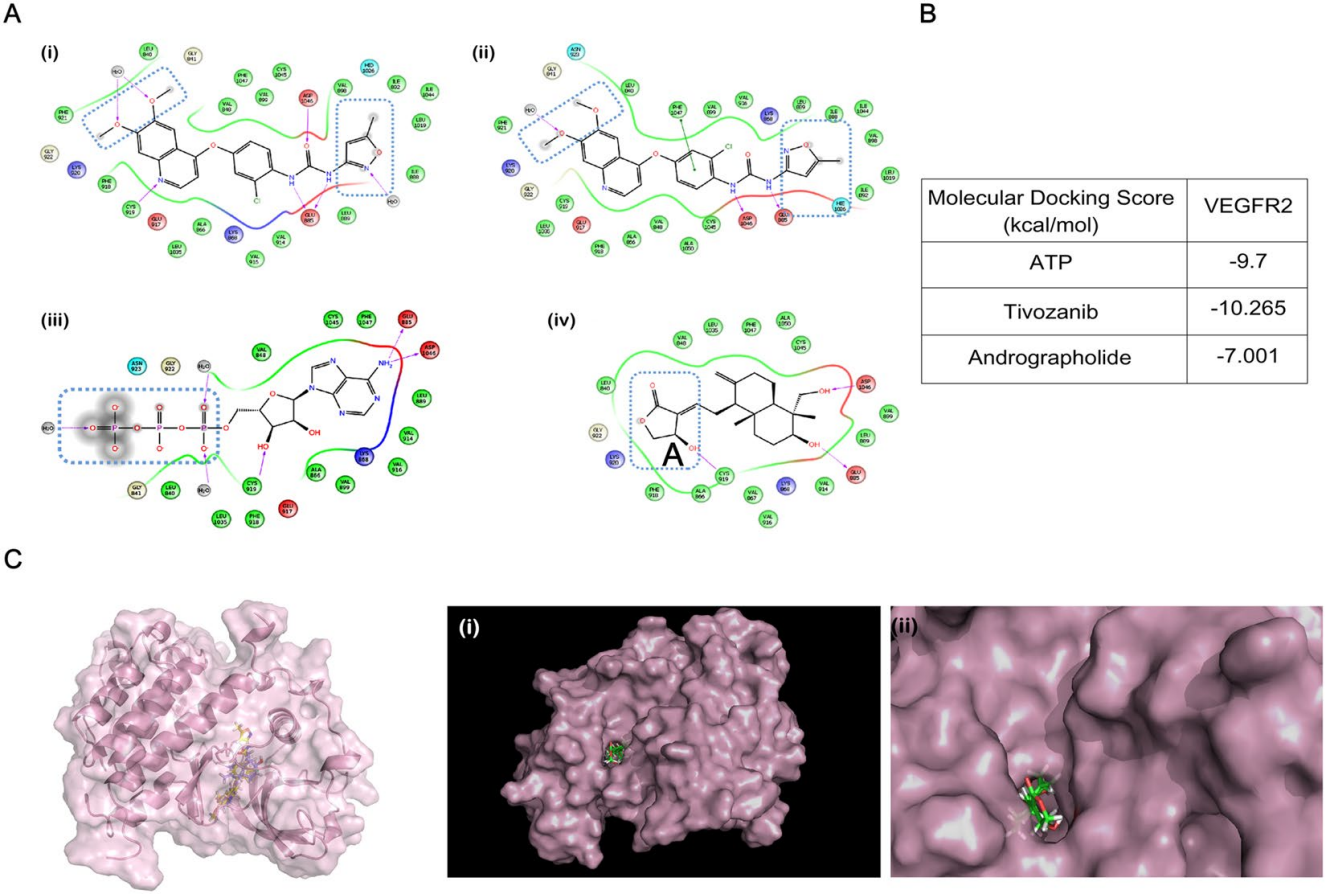
Andrographolide and tivozanib interacts with common amino acid in the ATP-binding pocket of VEGFR2. (**A**) 2D binding poses of ligands (i) co-crystal conformation of tivozanib with VEGFR2 (PDB ID: 4ASE), (ii) docked conformation of tivozanib in the kinase domain of VEGFR2, (iii) docked conformation of ATP in the kinase domain of VEGFR2 and (iv) docked conformation of andrographolide in the kinase domain of VEGFR2. All the ligands share common interacting amino acid Asp-1045 and Glu-885. (**B**) Molecular docking score of ATP, tivozanib, and andrographolide in ATP kinase domain of VEGFR2. (**C**) Surface model depicts andrographolide in kinase domain of VEGFR2, (i & ii) andrographolide is buried inside the ATP-binding pocket, protein is represented in the surface form, and andrographolide is represented in the stick form. (iii) Binding pocket is magnified for further understanding.

VEGFA secreted by tumor cells, bind to VEGFR2 and promote neo-vascularization. So, to inhibit the neo-angiogenesis we searched for a small molecule inhibitor which can potentially interact with the VEGFR2 kinase domain and inhibits its activity. Structures of VEGFR2 along with co-crystal ligand molecule were obtained from PDB databank (4ASE)^14^. 2D co-crystal-binding conformation of VEGFR2-tivozanib was shown in (Fig. 1A(i)). For benchmark study, tivozanib was docked in the ATP-binding pocket of VEGFR2 using Grid-based ligand docking with energetics (GLIDE) module of Maestro and obtained a docked conformation similar to the co-crystal-binding conformation (Fig. 1A(ii)). Docked conformation of ATP bound to VEGFR2 was also shown in (Fig. 1A (iii)). Using the same algorithms, a small molecule library was screened in the Schrodinger suit. Docked pose (Fig. 1A(iv)) and docking score Fig. 1B predicted that andrographolide has significant binding affinity with VEGFR2 as compared to ATP. It is a matter of great significance that tivozanib, ATP, and andrographolide share common interacting amino acids, i.e., Glu-885 and Asp-1046 in the kinase domain of VEGFR2. From both co-crystal and docked conformation of tivozanib it is seen that water molecules are imparting key interactions as highlighted by blue dotted lines (Fig. 1A(i),(ii)). Thus these water molecules were kept intact in the Grid generation. Same Grid was applied to docked ATP (Fig. 1A(iii)) and andrographolide (Fig. 1A(iv)) in the kinase domain of VEGFR2. Also, water molecules are found to be present in the vicinity of all the ligands (as highlighted in blue dotted lines). Ribbon and surface filling model of VEGFR2 showed that andrographolide (stick form) fits very tightly into kinase pocket of VEGFR2 (Fig. 1C).

### Molecular dynamic simulation and docking study confirms that VEGFR2-andrographolide association is stable under physiological condition

**Figure 2:**
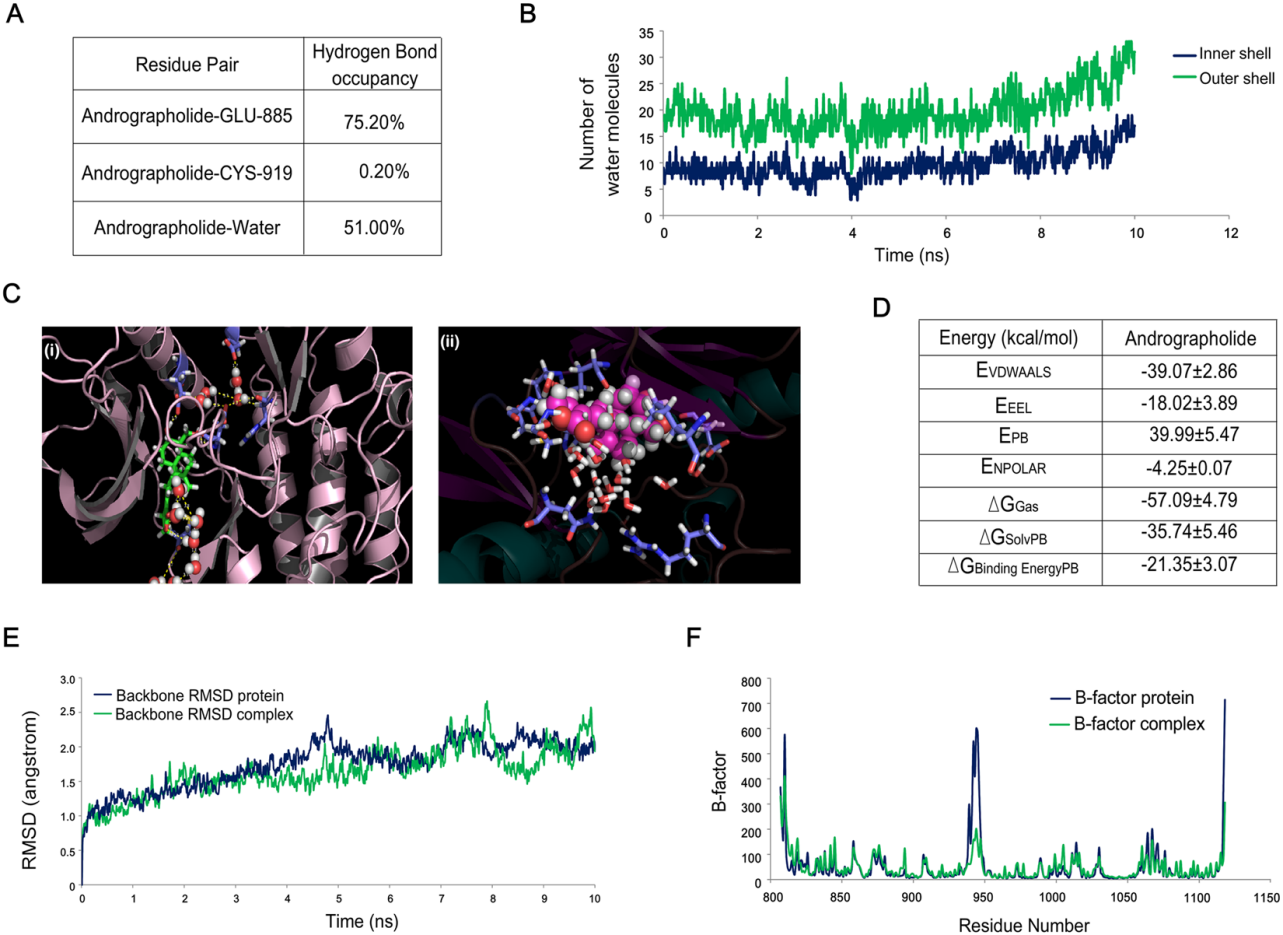
Molecular dynamic simulation and docking study confirms that VEGFR2-andrographolide association is stable under physiological condition. (**A**) Hydrogen bond occupancy of andrographolide with Glu-885 and Cys-919 amino acid present in the VEGFR2 kinase domain. Andrographolide interaction with hydrogen of surrounding water molecules has also been represented here. (**B**) The graph represents inner shell of water molecules which is at a 3.4Å distance and second solvation shell is at a 5Å distance from the ligand. (**C**) Ribbon model of VEGFR2, stick form is andrographolide and sphere models are water molecules. Crystalline water molecules were kept intact during the molecular docking andrographolide, docked conformation suggests that water-mediated interactions are assisting binding of andrographolide. (**D**) Binding free energy component of andrographolide with VEGFR2 based on MMPBSA calculations. Abbreviations mentioned above denotes following- E_VDWAALS_: Non-Bonded van der Waals Energy. E_EEL_: Non-Bonded Electrostatic Energy. E_PB_: Polar solvation energy (PB). E_NPOLAR_: Nonpolar solvation energy from repulsive solute-solvent interactions (PB). ∆G_Gas_: E_VDWAALS_ + E_EEL_ + Internal Energy. ∆G_Solv:_ Polar solvation energy + Non polar solvation energy. ∆G_Binding Energy_: Binding Free Energy (∆G_Gas_ + ∆G_Solv_). (E) Root mean square displacement (RMSD) of VEGFR2 (protein) and VEGFR2-andrographolide. (F) B-factor analysis suggests flexibility of protein decreases after interaction with andrographolide.

Hydrogen bond analysis suggests that andrographolide is forming hydrogen bond primarily with Glu-885 with high occupancy value and andrographolide is majorly supported by polar interactions with water molecules (Fig. 2A). These results correlate with water-shell analysis (Fig. 2B), number of water molecules surrounding ligand molecule are increasing with the progress of simulation. Inner shell indicates distance of 3.4Å from ligand and second solvation shell is the distance of 5Å from ligand. Molecular docking represents that seven water molecules from the front and five water molecules from the back help andrographolide to fit into kinase domain pocket of VEGFR2 (Fig. 2C(i,ii)). Binding free energies of andrographolide were estimated using Molecular Mechanics Poisson-Boltzmann Surface Area (MMPBSA) approach, over last 2 ns simulation frames with all default parameters of MMPBSA module of Amber (Fig. 2D). Result suggests that conformational stability of VEGFR2-andrographolide is energetically favorable due to electrostatic and Van der Waals interaction. To further gain the mechanistic insights of binding behavior of andrographolide with VEGFR2 an all atom molecular dynamics simulation study was conducted over the docked complex. The simulated trajectory was analyzed further. Root mean square displacement (RMSD) and B-Factor analysis determines the flexibility and stability of the system. As seen in (Fig. 2E), backbone RMSD of VEGFR2-andrographolide complex and unbound VEGFR2 is very low (~1.5–2Å), this infers that during simulation run the overall structure is not varying much from that of the starting conformation and system is stable throughout the simulation. B-Factor analysis suggests that flexibility of overall structure is reduced upon binding of andrographolide (Fig. 2F). Further to confirm drug like activity of andrographolide, absorption, distribution, metabolism and excretion (ADME) property was studied using QikProp module of Maestro^15^ Results suggest that andrographolide is bioavailable as it has drug like ADME coefficients (Table 1).

**Table 1:** ADME properties of andrographolide.

SN. No	S	MW	QPlogPo/w	QPlogS	QPPCaco	QPlogBB	PHOA	RO5
Andrographolide	0	350.454	1.459	−2.942	201.138	−1.343	76.719	0

ADME properties of andrographolide. ADME coefficients are - S (STARS) = Number of property/descriptor values falling outside the 95% range of similar values for known drugs. Recommended value 0–5. MW = Molecular weight. QPlogPo/w = Predicted octanol/water partition coefficient. Recommended values − 2.0–6.5. QPlogS = Predicted aqueous solubility, log S. Recommended values − 6.5–0.5. QPPCaco = Predicted apparent Caco-2 cell permeability in nm/sec. Recommended values <25 poor, >500 great. QPlogBB = Predicted brain/blood partition coefficient. Recommended values − 3.0–1.2. PHOA = Predicted human oral absorption on 0 to 100% scale. Recommended values >80% is high < 25% is poor. RO5 = Rule of Five, The rules are: mol MW <500, QPlogPo/w <5, donor HB ≤5, and accpt HB ≤10.

### Andrographolide inhibits neo-angiogenesis

**Figure 3:**
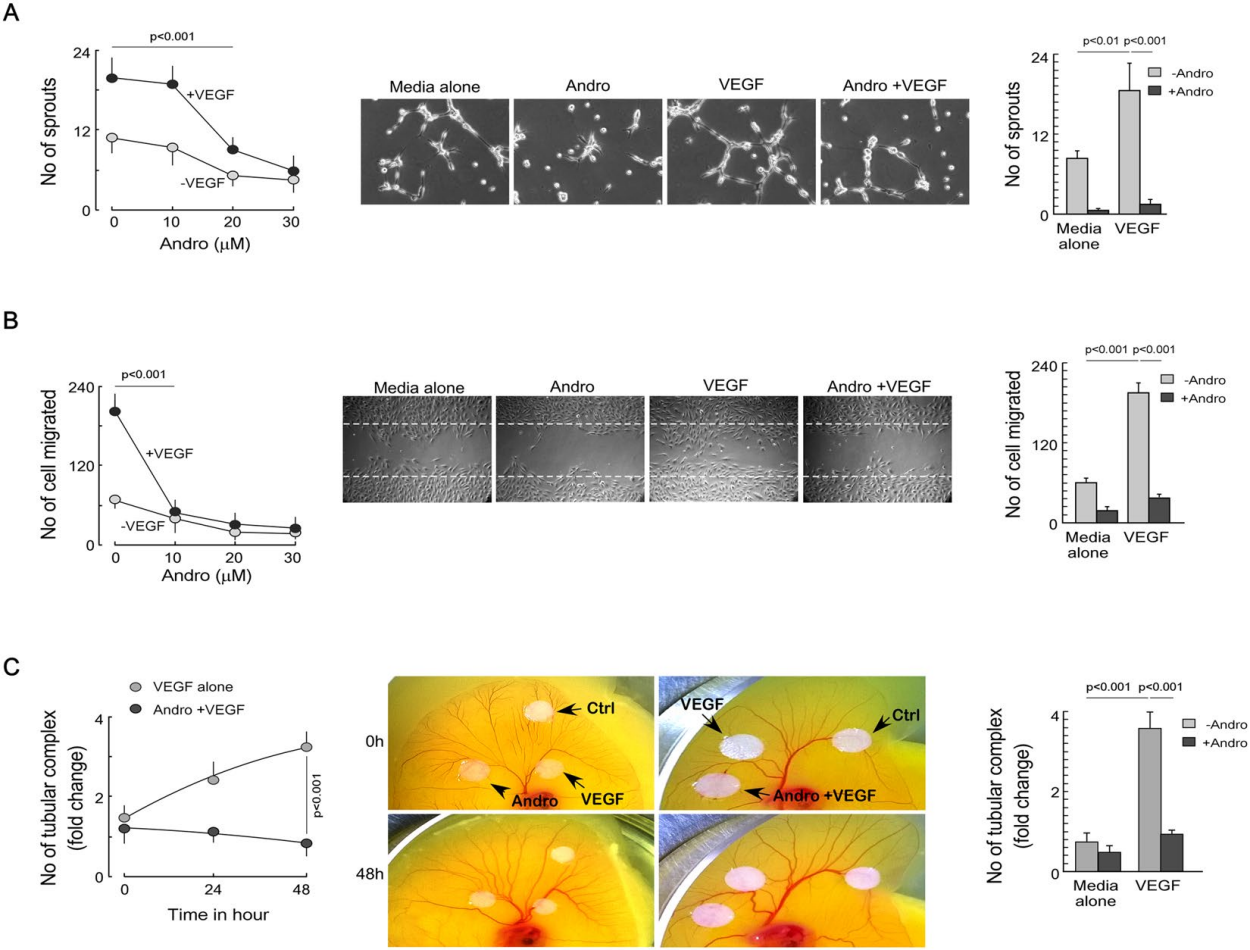
Andrographolide inhibits neo-angiogenesis. (**A**) Matrigel-embedded endothelial cells pretreated with different doses of andrographolide were further stimulated with 10 ng/ml VEGF. Number of sprouts formed were quantified and graph was plotted (*left*). Matrigel-embedded endothelial cells were pretreated with 20 µM andrographolide were stimulated with or without 10 ng/ml VEGF. Images were taken after 4–6 h (*middle*). Number of sprouts formed were plotted graphically (*right*). (**B**) Andrographolide-pretreated endothelial cells were treated with VEGF and number of migrated cells were quantified and plotted graphically (*left*). Wound healing assay was performed with VEGF presence of 20 µM andrographolide (*middle*). The number of cells migrated in wound area were counted and plotted graphically (*right*). (**C**) Chick embryo was treated with 20 µg andrographolide for different time intervals and number of tubular complex formed in presence and absence of VEGF were assayed by Angioquant software (*left*). CAM assay was performed in presence or absence of VEGF (*middle*). The number of tubular complexes were quantified using Angioquant software and plotted graphically (*right*). Values are mean ± SEM of three independent experiments in each case or representative of a typical experiment.

*In-silico* study revealed that andrographolide fits very nicely into kinase pocket of VEGFR2. It is, therefore, hypothesized that andrographolide binds to kinase domain and inhibit VEGFR2 activation and neo-angiogenesis in the tumor microenvironment. So, neo-angiogenesis assays were performed to validate the anti-angiogenic effect of andrographolide. Human umbilical vein endothelial cells (HUVECs) embedded in matrigel were treated with andrographolide (20 µM) in presence or absence of VEGFA (10 ng/ml). The number of sprouts formed (Fig. 3A) and endothelial cell migrated in wound area were quantified by Image-J software (Fig. 3B). It was observed that andrographolide significantly inhibits the VEGFA-induced sprout formation and cell migration (Fig. 3A,B). In support of the previous results, chorio-allantoic membrane (CAM) assay was performed to study the *ex-vivo* effect of andrographolide on VEGFA-induced new blood vessel formation. Three-day-old fertilized egg was treated with VEGFA presence and absence of andrographolide (Fig. 3C *left* and *middle panels*) and allowed to develop new blood vessel. Angioquant image analysis showed that andrographolide significantly regresses VEGFA-induced neo-angiogenesis in chick embryo (Fig. 3C *right panel*)^16^. All these results certainly signify that andrographolide effectively inhibits neo-angiogenesis.

### Andrographolide inhibit activation of downstream signaling molecules

**Figure 4:**
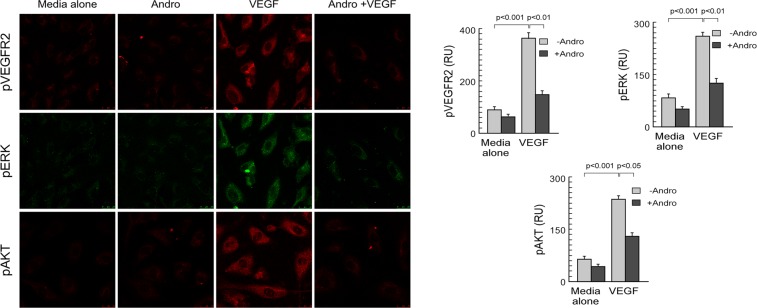
Andrographolide inhibit activation of downstream signaling molecules. The confocal microscopic study shows that 20 µM andrographolide inhibits VEGFA-induced phosphorylation of VEGFR2/ERK/AKT (*left panel*). Image magnification: 60x; scale bar: 10 µm. Mean fluorescence intensity was quantified using Image-J software and represented graphically (*right panels*). Values are mean ±SEM of three independent experiments in each case or representative of a typical experiment.

*In-silico* data indicated that andrographolide competes with ATP for binding to VEGFR2 kinase domain, which prompted us to assume that VEGFR2 phosphorylation and activation of downstream signaling molecules will be aborted. To check our assumption, endothelial cells were pre-treated with andrographolide followed by activation with VEGFA. The phosphorylation status of VEGFR2, extracellular signal-regulated kinase (ERK) and AKT were studied by confocal microscopy (Fig. 4, *left*) and represented graphically (Fig. 4, *right*). Results showed that andrographolide significantly perturbed VEGFA-downstream signaling.

### Andrographolide inhibits neo-angiogenesis in the tumor site

**Figure 5:**
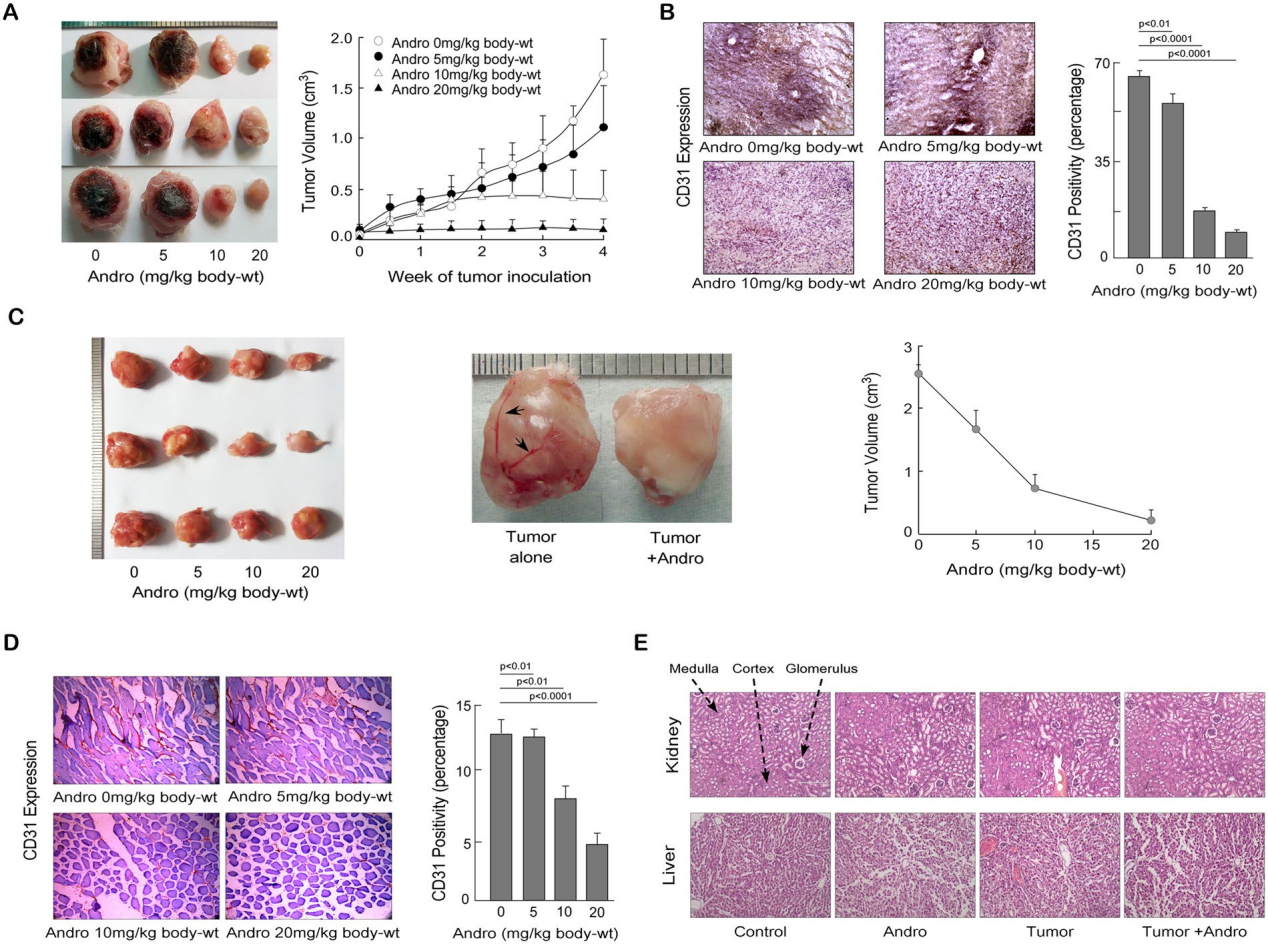
Andrographolide inhibits neo-angiogenesis in tumor site. (**A**) Isogenic mouse breast tumor (4T1 cells) were implanted in the mammary pad of BALB/c mice. Various doses of andrographolide reduced the blood vessel formation, tumor size (*left*) (ii) Tumor volume in 4T1 implanted mice were measured and plotted graphically (*right*). (**B**) Immunohistological images showing the expression level of CD31-positive cells in the mice breast tumor tissue. Area of tumor section expressing CD31 was analyzed using Image-J software, represented graphically. (**C**) Andrographolide inhibits tumor size and tumor volume in multi-drug resistant S-180-implanted tumor in Swiss albino mice (*left* and *middle*). Arrow-head showing the new blood vessel in tumor site (*middle*). Tumor volume in S-180-implanted mice were measured and plotted graphically (*right*). (**D**) The expression level of CD31-positive cells in andrographolide-treated S-180-implanted tumor. Area of tumor section expressing CD31 was analyzed using Image-J software and represented graphically. (**E**) Kidney and liver sections (4T1-injected mice) were stained with haematoxylin and eosin. Values are mean ±SEM of five independent experiments in each case or representative of a typical experiment.

*In-vitro* data indicate that andrographolide efficiently inhibits neo-angiogenesis. To validate its efficacy in *in-vivo* tumor condition, various doses of andrographolide were fed orally to breast tumor (4T1)-implanted BALB/c mice. The tumor volume was measured at 4^th^ week of tumor inoculation. The maximum reduction in tumor load was noticed with the andrographolide treatment at a dose of 10 mg/kg body-weight with concomitant reduction of CD31 expression (endothelial cell marker) which further correlated with the decrease in new blood vessel formation. Expression of CD31 was quantified using Image J software and graph was plotted (Fig. 5A,B). In parallel sets, multi-drug resistant S180 cells were injected in right thigh-pad of Swiss albino mice to study the effect of andrographolide in this system. Similar to breast tumor model this tumor which is difficult to treat, also showed a significant reduction in tumor volume (Fig. 5C); and number of blood vessels as a result of andrographolide treatment. Expression of CD31 was quantified using Image J software and graph was plotted (Fig. 5C, *right* & Fig. 5D). In order to confirm that 10 mg/kg body-weight of andrographolide is non-toxic, kidney and liver tissue sections from BALB/c mice were stained with haematoxylin and eosin. Tissue morphology which gets disrupted during tumor condition maintain its original architecture after andrographolide treatment (Fig. 5E).

All these data certainly signifies that andrographolide efficiently reduce tumor load, new blood vessel formation, and tumor volume and can be used as an anti-angiogenic agent.

## Discussions

Angiogenesis is a controlled series of event which involves the formation of new blood vessels from pre-existing vessels^4^. When tumor size exceeds beyond a defined size; lack of blood and oxygen supply and nutrient deficit condition prompts angiogenic-switch from vascular quiescence^6^. This angiogenic-signal leads to the release of VEGFA. VEGFA-VEGFR2 interaction causes receptor activation leading to the tyrosine kinase activation and switch-on of downstream signaling molecules which mediates endothelial cell migration, proliferation, survival, and vascular permeability^7,8^. Tyrosine kinases are principal proteins through which tumor cells manifests their purpose of cell survival, proliferation, angiogenesis and metastasis. Hence, these kinases are potential target for computer-based drug design to treat cancer. Computer aided studies provide information at molecular and atomic level which is impossible to achieve through wet-lab experiments^17^.

Hence, molecular modelling and simulation profiling is used for development of novel cancer drugs. Ligprep, GLIDE and Prime module of Maestro generated models which were further checked for binding affinity with VEGFR2 tyrosine kinase. Crystal structure of tivozanib in VEGFR2 kinase domain was taken from protein data-bank. Both tivozanib and andrographolide (*in-silico* chosen compound) was docked using GLIDE module and docking score was calculated. Molecular docking offered probable binding conformations of ligand molecules with ATP-binding site of VEGFR2. Both tivozanib and andrographolide are mimicking the binding pattern of ATP. Thus, like tivozanib, andrographolide can also act as a competitive inhibitor of ATP to VEGFR2. Here all the three molecules (ATP, tivozanib, andrographolide) are in vicinity of adenine pocket (Glu-917 and Cys-919) and back hydrophobic pocket (Glu-885 and Asp-1046), these are the key residues preferably targeted in the concerned drug design^18^. Negative docking score indicates binding of ligand is energetically favorable, known ligands ATP and tivozanib are showing lower docking energies than that of the andrographolide this might be due to lack of aromatic interactions which are found in tivozanib-Phe-1047. However, andrographolide is interacting with key residues Cys-919, Glu-885 and Asp-1046 thus, we can estimate that andrographolide binding with VEGFR2 is feasible.

Molecular simulation application involves dynamic processes where molecule is represented as collection of atoms. The potential energy of system is based on set of equations and parameters, which are collectively known as *force field*. The result of molecular simulation experiment is generated as a set of particle velocities and coordinates which are generally termed as trajectories. These trajectories provide information about presence of hydrogen bonds, distance between atoms etc.^17^. In our simulation experiments Amber14 force field was used. Hydrogen bond analysis suggested that hydrogen bonds present in water molecules provide anchorage to andrographolide in kinase domain. Hydrogen bond analysis, binding free energy, RMSD and B-factor result simultaneously deduce that association between VEGFR2 and andrographolide physiologically stable.

Andrographolide is highly bioavailable (Table 1) and has no side effects (Fig. 5E). Andrographolide has been widely used as an anti-inflammatory and hepatoprotective drug^10^ for centuries. Along with *in-silico* studies we performed wet-lab experiments to validate efficacy of andrographolide as anti-angiogenic compound. Several experiments were designed to study effect of andrographolide over VEGFA-induced neo-vascularization. Sprout formation, wound-healing assay in endothelial cells and CAM assay are well established *in-vitro* and *ex-vivo* assays to study the neo-angiogenesis. Andrographolide impede VEGFA-induced sprout formation, cell migration in endothelial cells and retard growth of new blood vessels in chick embryo. During neo-angiogenesis secreted VEGFA binds to VEGFR2 on the endothelial cell surface. This event leads to phosphorylation of VEGFR2 and activation of downstream signaling molecules like ERK and AKT which triggers proliferation and migration of endothelial cells. In accordance with our *in-silico* finding, andrographolide compete with ATP for kinase domain in VEGFR2 hence doesn’t allow tyrosine kinase activity which blocks phosphorylation and activation of downstream signaling molecules like ERK and AKT. For translational purpose we used two different mice model e.g., (i) syngeneic breast tumor and (ii) multi-drug resistant sarcoma mice models. *In-vivo* experiments showed that andrographolide inhibits VEGFA-mediated neo-angiogenesis. Andrographolide at non-toxic dose suppress tumor-angiogenesis. Both the chemotherapy-sensitive breast tumor and chemotherapy-resistant sarcoma model confirmed that andrographolide is effective irrespective of the type of tumor.

## Methods

### Cell culture

Human umbilical vein endothelial cells (HUVECs) were purchased from HiMedia (Proforma Inv. No: 000075), India. HUVEC cells were cultured in HUVEC growth supplemented media (HiMedia). Media was further supplemented with 5% heat-inactivated FBS (Lonza, USA), penicillin, streptomycin, amphotericin B. Cells were maintained in a humidified atmosphere of 5% CO_2_ at 37 °C.

### Cell viability assay

Cells were grown to confluence in gelatin-coated 24-well plate. Serum starved cells were pre-treated (90 min) with or without andrographolide. Andrographolide was washed off and then cells were treated with VEGF (10 ng/ml) for 18 h. Viable cells were counted using trypan-blue dye exclusion test by haemocytometer under microscope, 10x magnification. Viable cells were colorless while the dead cells were blue. Viable cells were plotted as percentage of control.

### Purification of Andrographolide from the Aerial parts of Andrographis paniculata

Organically grown *Andrographis paniculata* plant was obtained from Jeevaniya Society, Lucknow, India and identified by CSIR-NISCAIR Herbarium and Museum, New Delhi. The aerial parts of the plant was removed, washed with distilled water and dried in an oven at 60 °C overnight and converted into powder by pulverization. 100 gm of the powder was extracted with 1 litre of Ethanol: Water (1:1 v: v) at 40 °C in a water bath shaker overnight. The extract was filtered and the clear filtrate was evaporated in a rotary evaporator at reduced pressure to 100 ml of slurry. 20 ml of this slurry was loaded on to a silica gel (Merck, India, 60–120 mesh) column (20 cm × 2.5 cm) using hexane and eluted sequentially with 200 ml each of hexane followed by analytical-grade ethyl acetate, acetone and finally with methanol. Each solvent fraction was evaporated and the residual matter was examined by TLC by comparing with authentic andrographolide (Sigma Aldrich-USA). The material present in the acetone fraction was crystallized from methanol to give yellow crystals which was identified to be andrographolide on the basis of its thin layer chromatography (TLC) profile, melting point, and High Performance Liquid Chromatography (HPLC) and ^1^H NMR profiles and compared with authentic andrographolide (Sigma Aldrich-USA).

### Animal model

To deduce effective and non-toxic anti-angiogenic dose of andrographolide BALB/c and Swiss albino mice were used. Mice (NCLAS, Hyderabad, India) weighing 20–25 g were maintained in a temperature-controlled room with light-dark cycle. All animal experiments were performed following Principles of laboratory animal care (NIH publication No. 85–23, revised in 1985) as well as Indian laws on ‘Protection of Animals’ under the provision of the Bose Institute Ethics Committee for the purpose of control and supervision of experiments on animals (Reg. No. 95/99/CPCSEA; Approval No: IAEC/BI/24/2015). 2 × 10^6^ 4T1 (ATCC) cells were inoculated in the mammary fat-pad of BALB/c mice. 1.5 × 10^6^ multi-drug resistant S180 (ATCC) cells were inoculated in thigh pad of Swiss albino mice. Tumor-bearing mice was divided into 4 groups with 5 mice in each set; (i) control, (ii) 5 mg/kg body-weight, (iii) 10 mg/kg body-weight, and (iv) 20 mg/kg body-weight andrographolide. After seven days of tumor inoculation, andrographolide was fed orally to mice every alternate day. Mice were observed up to 28 days. Tumor volume was measured every alternate day. Mice were sacrificed after 28 days and images were taken before and after the sacrifice. Tumor weight and volume were taken after mice sacrifice. Tumor tissue was fixed in Bouin’s solution for immunohistochemistry study.

### Wound-healing assay

Migration of human HUVEC was determined using wound-healing assay. Cells were grown to confluence in gelatin-coated 12-well plates. Sterile microtip was used to create a wound on a monolayer of serum-starved cells pre-treated (90 min) with or without andrographolide. Then cells were treated with recombinant VEGF (10 ng/ml). HUVEC monolayer cells were incubated for 18 h and images were captured under the inverted phase-contrast microscope (Olympus IX71). The number of cells migrated to the wound area was calculated by Image-J software^19,20^.

### Sprout formation assay

The spontaneous formation of vessel‐like structures by endothelial cells was performed on a basement membrane matrix preparation (Matrigel, BD Biosciences, and USA). Plates were coated with Matrigel (20 mg/ml), and the surface was allowed to polymerize at 37 °C for 1 h. HUVECs were seeded over Matrigel-coated wells, incubated for 1 h at 37 °C. Further cells were treated accordingly, and images were captured after 4–6 hours by inverted phase-contrast microscope, and the tube forming intact network was counted^20^.

### Chorioallantoic membrane (CAM) assay

CAM assay is the most widely used *ex-vivo* assay used for studying neo-angiogenesis. The three-day-old fertilized egg was broken to keep embryo intact. Treatment was done according to experimental requirement. The egg was monitored at a regular interval, and images were taken. Images were quantified using Angioquant software, an automated tool to quantify angiogenesis (http://www.cs.tut.fi/sgn/csb/angioquant/)^16^.

### Confocal microscopy

HUVEC (seeded over coverslip) were fixed, permeabilized and blocked with 3% BSA. Primary antibody (1:100) were diluted in 1% BSA in PBS and incubated for overnight at 4 °C. Cells were counterstained with fluorescent-tagged pVEGFR2 (Tyr-1175, Sigma USA), pAKT (Ser-473, Cell Signaling, USA) and/or pERK (Tyr-204, Santa Cruz Biotechnology, USA) antibodies. Cells were mounted with DPX and imaged with Olympus inverted confocal microscope using 63x/100x objectives. Laser intensities and detector gains were maintained at the same level during all imaging sessions for each experiment. Image analysis between pixels of different channels have been done using Image-J software^21^.

### Immunohistochemistry

Tissues were dissected out; fixed in Bouin’s fixative overnight, cryo-protected and serial sections were cut on a cryostat (CM1850; Leica) at 4–5 μm thickness. The tissue sections were washed with PBS, antigen retrieval was done with enzymatic retrieval method using trypsin-CaCl_2_ solution. Sections were blocked with 1% BSA at 37 °C. Sections were incubated overnight at 25 °C in a humid atmosphere with primary antibodies against CD31 (1:100; Santa Cruz Biotechnology, Inc.). The sections were and then incubated with biotinylated anti-mouse IgG followed by peroxidase conjugate extra-avidin (Sigma-Aldrich; 1:100) for 60 minutes and 3,3′-diaminobenzidine was used as chromogen (Sigma-Aldrich AEC101-1KT; 1:100) to visualize the reaction product and counterstained with hematoxylin (1:1; Himedia, India). Finally, sections were washed in distilled water and mounted in glycerol gelatin. Kidney and liver sections were cut on a cryostat at 4–5 μm thickness. Sections were stained with haematoxylin and eosin. Images were acquired with a bright-field microscope (Leica) at 10x magnification.

### Structural Biology

The 3D structure of andrographolide and ATP were built in Maestro^22^ and further plausible conformations of ligands were generated using LigPrep^23^ module of Maestro. Receptor structure of VEGFR2 (4ASE)^14^ were obtained from PDB databank. Receptors were prepared for further molecular docking analysis of ligands under study using protein preparation wizard^24^ of Maestro. Grids were generated over the binding sites of co-crystal ligands with the inner box size of 10Å and outer box size of 30Å. The grid of VEGFR2 was validated by docking of co-crystal ligand, *i.e*., tivozanib, the co-crystal interaction pattern of the ligand was conserved in the docked conformation. Tivozanib is already found as an inhibitor of VEGFR2^14^. Ligands were docked using Grid Based Ligand docking, i.e., GLIDE module of Maestro with standard precision mode^25^. Here, various predicted docked conformations of ATP and andrographolide with ATP-binding domain of VEGFR2 are overlapping with the top-most docked pose (based on docking score) which indicates that the best-docked pose might represent the feasible bioactive conformation. Docked complex with best docking score of andrographolide-VEGFR2 was considered for further molecular dynamics simulation analysis. Also unbound state of VEGFR2 was taken as a control system. The missing residues in the PDB structure of VEGFR2 (VAL990, ALA991, PRO992, GLU993, ASP994 and, LEU995) were built and refined using PRIME module of Maestro^26^.

Simulation analysis was performed with AMBER14^27^. The simple harmonic function used by General Amber Force Field (GAFF) and AM1-BCC charge method was used for parameterization of andrographolide and protein system with ff99SB and ff99SBildn force-fields of AMBER14^28,29^. Systems were neutralized with counter-ions and further solvated with 8Å water box of TIP3P water model. The standard protocol of AMBER simulation was followed two-step minimization, (i) heating for 50 ps, and (ii) equilibration phase for 1 ns. Equilibrated systems were used for a further production run of 10 ns on an NPT ensemble at a 300 K temperature and 1 atm pressure, with a step size of 2 fs. Langevin thermostat, barostat were used, and SHAKE algorithm was applied^30,31^. Particle Mesh Ewald method was applied for long-range electrostatic interactions with 0.1 nm grid space of fast Fourier transform grid, and the non-bonded cut off was kept at 12Å^32^. Coordinates are recorded in trajectory at each 10 ps time step. Ptraj module of AMBER, PyMol and VMD are used for the analysis and visualization purposes^33–35^. Binding free energies of andrographolide were estimated using Molecular Mechanics Poisson-Boltzmann Surface Area (MMPBSA) approach over last 2 ns simulation frames with all default parameters of MMPBSA module of amber^36–39^.

### Statistical analyses

Values are shown as the standard error of mean (SEM) except when otherwise indicated. Comparison of multiple experimental groups was performed by I‐way ANOVA test followed by Bonferroni post‐hoc Test. Data were analysed and, when appropriate, the significance of the differences between mean values was determined by a Student’s t-test. Results were considered significant at p < 0.05.

## Data Availability

Materials, data and associated protocols will be available on request.
